# A Rare Case of a Large Diaphragmatic Hernia With Upward Hepatic Transposition

**DOI:** 10.7759/cureus.100034

**Published:** 2025-12-24

**Authors:** Roberto F de Oliveira, André Henrique V de Almeida, Leonardo F Mello, Ariana Albertina A Leal, Danila F Silva, José Aderval Aragão, Bento João Abreu

**Affiliations:** 1 Human Anatomy Division, School of Medicine, Centro Universitário de Excelência (UNEX), Feira de Santana, BRA; 2 Department of Human Anatomy, School of Medicine, Faculdade da Região Sisaleira (FARESI), Conceição do Coité, BRA; 3 Department of Anatomy, Universidade Tiradentes, Aracaju, BRA; 4 Department of Morphology and Medicine, Federal University of Sergipe (UFS), Aracaju, BRA; 5 Department of Morphology, Federal University of Rio Grande do Norte, Natal, BRA; 6 Department of Clinical Anatomy, VARIANTIS Research Laboratory, Płock, POL

**Keywords:** anatomical variation, anomaly, diaphragm, hernia, liver, thoracic cavity

## Abstract

This study reports a rare case of an intrathoracic hepatic hernia identified during cadaveric dissection, characterized by a large congenital hiatus in the right diaphragmatic dome that permitted the herniation of the right hepatic lobe and displacement of adjacent abdominal viscera. The case was observed in the cadaver of an adult male, approximately 50 years old. During anatomical dissection, a defect measuring about 13 cm in the right dome of the diaphragm was found, resulting in the migration of the entire right hepatic lobe into the thoracic cavity, compression of the right lung, mediastinal shift, and displacement of other viscera, including splenic ectopia and migration of the hepatic flexure and transverse colon. The anatomical findings were documented photographically, measured, and analyzed in light of the existing literature. Intrathoracic liver hernias pose diagnostic challenges because they may mimic mediastinal or pulmonary masses. This case highlights the extensive thoracoabdominal reorganization that can result from congenital diaphragmatic defects and emphasizes the importance of thorough anatomical and imaging evaluations to ensure accurate diagnosis and to guide clinical and surgical management.

## Introduction

Diaphragmatic hernias are uncommon clinical conditions in which abdominal organs protrude into the thoracic cavity due to a defect or loss of integrity of the diaphragm. With a reported prevalence ranging from 0.7 to 15.9 per 10,000 births [1], they are classified as either congenital or acquired [2]. These alterations compromise the integrity of the physical barrier between the thoracic and abdominal cavities, potentially leading to severe pathophysiological outcomes such as pulmonary compression and displacement of mediastinal structures [3]. Among these, intrathoracic hepatic hernias represent an even rarer condition, characterized by marked viscera displacement and greater management complexity [4].

The diaphragm forms between the fourth and 12th weeks of embryogenesis through the fusion of the transverse septum, pleuroperitoneal folds, esophageal mesentery, and muscular body wall. Proper integration of these structures, including lateral fusion with the body wall and posterior fusion with the esophageal mesentery and pleuroperitoneal folds, is essential for the formation of an intact diaphragm. Disruptions in these fusion processes during development can result in defects of diaphragmatic integrity, allowing abnormal communication between the thoracic and abdominal cavities [5]. In this context, the etiopathogenesis of diaphragmatic congenital hernias is associated with disturbances in embryologic development, involving dysfunction in the closure of the pleuroperitoneal canal and in the formation of the posthepatic mesenchymal plate [2,6]. This type of defect often remains asymptomatic, even in the presence of precipitating factors such as increased intra-abdominal pressure, which may promote the migration of abdominal structures to the thorax, particularly the liver [7]. Recent studies have shown a forecast of poor prognosis in such cases, including complications such as liver congestion and pulmonary hypertension, which further hinder the diagnosis and therapeutic management in patients with large non-cirrhotic diaphragmatic defects [4,8].

Furthermore, acquired diaphragmatic hernias may result from post-operative states or chronic diseases that affect the diaphragm’s ability to stretch [9,10]. These situations may pose diagnostic challenges, and cases are sometimes misinterpreted as mediastinal masses or rare entities such as hepatopulmonary fusion [6]. Confirmation of diagnosis in such cases requires high-resolution imaging techniques, including CT scans and MRI, which are essential for accurately assessing visceral displacement and thoracic involvement of the condition [11,12].

The rarity of intrathoracic liver hernias underscores the need for precise anatomical investigation to clarify structural and vascular relationships and to determine their clinical significance. Despite recent diagnostic advances, including the use of artificial intelligence [11], reports describing complete displacement of the right hepatic lobe with major visceral reorganization remain scarce, highlighting the need for further detailed studies on this topic. In this study, we report a rare case of intrathoracic liver herniation. Moreover, morphofunctional alterations, including lung compression and mediastinal displacement, were examined, reinforcing the importance of meticulous anatomical studies for improving scientific knowledge and medical practice.

## Case presentation

**Figure 1:**
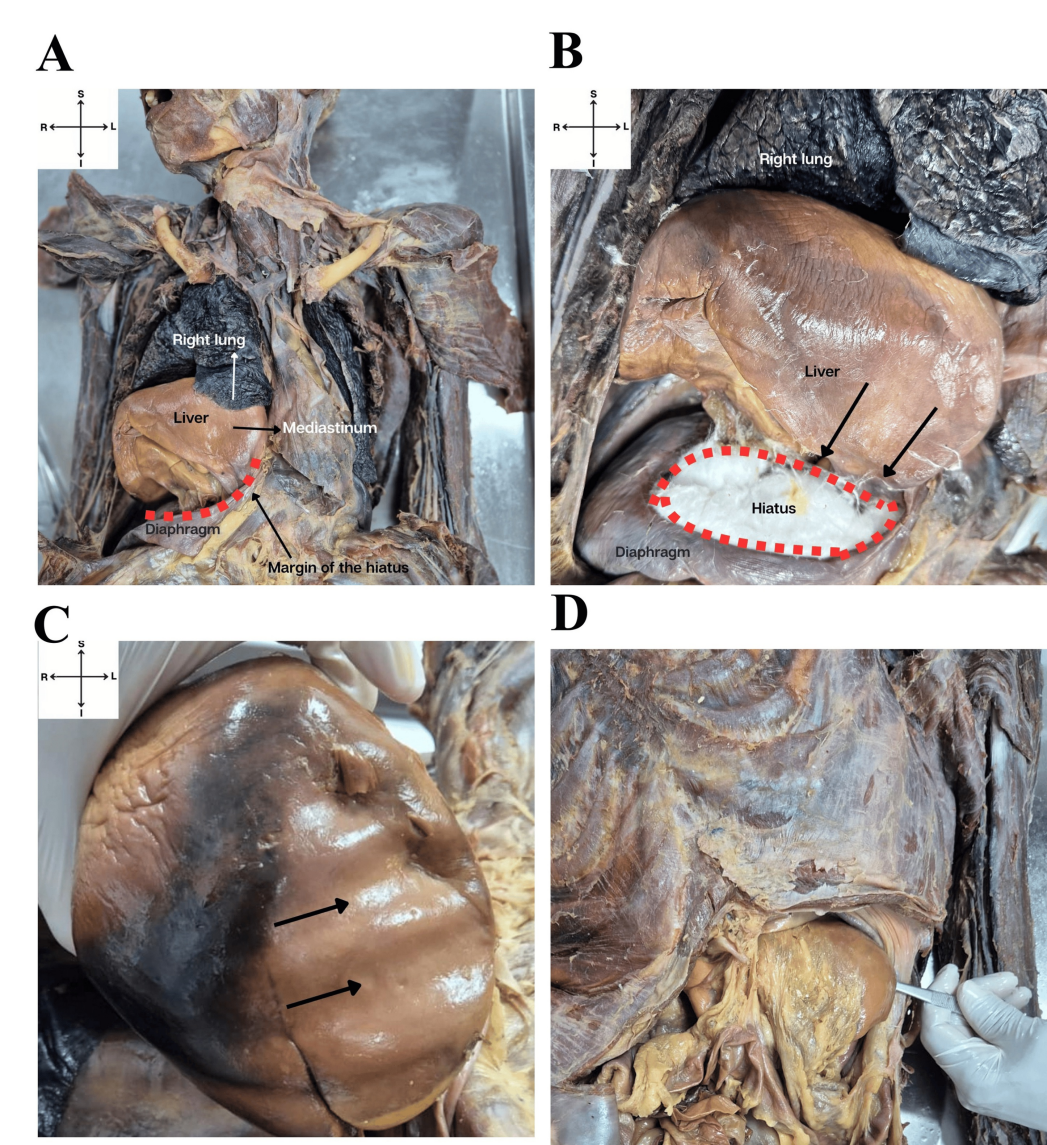
Overview of intrathoracic hepatic hernia. A) Thoracic cavity exposed, showing displacement of the right lobe of the liver into the thoracic cavity and its relationship with adjacent structures such as the right lung and mediastinum. B) The right liver lobe herniated through the diaphragm hiatus (outlined with red dashed lines), compressing the right lung. C) Impressions on the diaphragmatic surface of the liver, showing grooves (black arrows) of pressure adaptation of adjacent thoracic structures. D) The forceps highlight an elongated left hepatic lobe extending beyond the epigastric region of the abdomen.

During cadaveric dissection at the Anatomy Laboratory of the Centro Universitário de Excelência (UNEX), a rare intrathoracic hepatic herniation associated with a large congenital hiatus defect in the right dome of the diaphragm was identified in a 50-year-old male cadaver. This finding revealed a marked anatomical reorganization between the abdomen and thorax, with notable morphofunctional implications. The herniation resulted in the displacement of the liver into the thoracic cavity, causing compression and establishing new anatomical relationships with the ribs, mediastinum, and right lung (Figure 1A). The entire right liver lobe herniated through this defect into the thoracic cavity, lying beneath the right lower lung lobe and severely compressing it, likely impairing respiratory function (Figure 1B). Additionally, mediastinal displacement to the left was noted, altering the thoracic structure. The diaphragmatic surface of the liver was preserved but showed deep grooves, reflecting long-term adaptation to surrounding pressures (Figure 1C). These grooves indicate direct contact between the liver and thoracic structures, allowing gradual adjustment to mechanical forces. The observed hiatus measured approximately 13 cm in diameter and had well-defined, uniform edges, suggesting a congenital origin. An elongation of the left hepatic lobe was also observed (Figure 1D).

## Discussion

This study reports a unique case of intrathoracic liver herniation associated with a large congenital diaphragmatic defect, resulting in significant visceral displacement, including elongation of the left liver lobe, displacement of the hepatic flexure and transverse colon, and splenomegaly. These detailed observations contribute to understanding the morphofunctional consequences of large diaphragmatic hernias, representing a novel addition to the existing literature [8-11,13,14]. It is well known that the liver begins developing around the third to fourth week of gestation from the foregut endoderm as the hepatic diverticulum. During this period, the diaphragm forms from multiple components, including the septum transversum, pleuroperitoneal membranes, dorsal mesentery of the esophagus, and body wall musculature. By the eighth week, the diaphragm is largely complete, with the aortic hiatus forming posteriorly to allow passage of the aorta, thoracic duct, and azygos vein. Proper fusion of these structures is essential to maintain the integrity of the thoracoabdominal barrier. Embryonic alterations during this critical period can lead to aberrant conditions, such as the intrathoracic hepatic hernia described in the present case [15].

The marked pulmonary and mediastinal displacement observed here supports previous studies showing that large diaphragmatic hernias can reduce thoracic space and induce chronic lung remodeling [4,12]. This case demonstrates how thoracic migration of the liver can mechanically impair respiration and mimic clinical features seen in children with uncorrected congenital diaphragmatic hernias. The adaptation of the left liver lobe is particularly notable, as its elongation reflects long-term structural remodeling. This supports evidence that the liver has a remarkable capacity to adapt to chronic compression [8]. Moreover, the pronounced intestinal displacement observed in this case further highlights the impact of extremely large diaphragmatic hiatuses [9,14]. The presence of splenomegaly and an ectopic spleen in this case highlights a rarely discussed aspect of visceral displacement in diaphragmatic hernias. Previous studies indicate that significant organ displacement can affect not only the diaphragm and lungs but also neighboring abdominal structures, potentially causing systemic dysfunctions that remain poorly defined [16,17].

From a diagnostic standpoint, intrathoracic liver hernias are challenging because they can mimic mediastinal or pulmonary tumors [18]. Advanced imaging techniques, including automated MRI segmentation, have recently improved diagnostic accuracy and reduced errors, particularly in cases with nonspecific symptoms [10]. In this context, prenatal evaluation of diaphragmatic hernia using high-quality fetal ultrasound and magnetic resonance imaging is crucial for developing individualized prenatal and postnatal management strategies and for providing reliable prognostic expectations.

Reconstruction of major diaphragmatic defects requires personalized approaches. Cain-Trivette et al. used MRI-based compartmentalization to simplify imaging of the affected region [7], while Addeo et al. employed synthetic prostheses to reinforce the diaphragm and obtain detailed anatomical information, guiding therapeutic decisions in cases with substantial visceral displacement [19].

Embryological defects, such as incomplete closure of the pleuroperitoneal canal and abnormal formation of the posthepatic mesenchymal plate, underlie large diaphragmatic hiatuses [6,20]. Genetic factors may also contribute to prominent anatomical variations, linking the findings in this case to patterns observed in other studies [16].

This work has limitations. Given that this study was conducted on a donated cadaver, a principal limitation was the inability to evaluate the hemodynamic and respiratory implications of the observed anatomical variations, owing to the absence of functional and clinical assessments. Consequently, establishing a correlation between these structural alterations and their potential physiological or clinical significance was not feasible.

## Conclusions

This report describes a rare intrathoracic hepatic hernia with a large diaphragmatic hiatus, highlighting complex anatomical variations. Detailed anatomical analysis, combined with radiological and functional imaging, is crucial for accurate diagnosis and avoiding misinterpretation as pulmonary or mediastinal masses. Despite the lack of clinical and genetic data, these findings provide valuable insights for surgical planning and the management of diaphragmatic abnormalities.
